# Altered brain gene expression but not steroid biochemistry in a genetic mouse model of neurodevelopmental disorder

**DOI:** 10.1186/2040-2392-5-21

**Published:** 2014-03-06

**Authors:** Simon Trent, Jonathan P Fry, Obah A Ojarikre, William Davies

**Affiliations:** 1 Neuroscience and Mental Health Research Institute, Cardiff University, Cardiff, UK; 2 Department of Neuroscience, Physiology and Pharmacology, University College London, London, UK; 3 Medical Research Council National Institute for Medical Research, London, UK; 4 Medical Research Council Centre for Neuropsychiatric Genetics and Genomics, Cardiff University, Cardiff, UK; 5 School of Psychology, Cardiff University, Tower Building, Park Place, Cardiff CF10 3AT, UK

**Keywords:** Acetylserotonin O-methyltransferase, COUMATE, Steroid sulphatase, 39,X^Y*^O

## Abstract

**Background:**

The 39,X^Y*^O mouse, which lacks the orthologues of the ADHD and autism candidate genes *STS* (steroid sulphatase) and *ASMT* (acetylserotonin O-methyltransferase), exhibits behavioural phenotypes relevant to developmental disorders. The neurobiology underlying these phenotypes is unclear, although there is evidence for serotonergic abnormalities in the striatum and hippocampus.

**Methods:**

Using microarray and quantitative gene expression analyses, and gas chromatography–mass spectrometry, we compared brain gene expression and steroid biochemistry in wildtype (40,XY) and 39,X^Y*^O adult mice to identify non-obvious genetic and endocrine candidates for between-group differences in behaviour and neurochemistry. We also tested whether acute STS inhibition by COUMATE in wildtype (40,XY) adult male mice recapitulated any significant gene expression or biochemical findings from the genetic comparison. Data were analysed by unpaired *t*-test or Mann Whitney *U*-test depending on normality, with a single factor of KARYOTYPE.

**Results:**

Microarray analysis indicated seven robust gene expression differences between the two groups (*Vmn2r86*, *Sfi1*, *Pisd-ps1*, *Tagap1*, *C1qc*, *Metap1d*, *Erdr1*); *Erdr1* and *C1qc* expression was significantly reduced in the 39,X^Y*^O striatum and hippocampus, whilst the expression of *Dhcr7* (encoding 7-dehydrocholesterol reductase, a modulator of serotonin system development), was only reduced in the 39,X^Y*^O hippocampus. None of the confirmed gene expression changes could be recapitulated by COUMATE administration. We detected ten free, and two sulphated steroids in 40,XY and 39,X^Y*^O brain; surprisingly, the concentrations of all of these were equivalent between groups.

**Conclusions:**

Our data demonstrate that the mutation in 39,X^Y*^O mice: i) directly disrupts expression of the adjacent *Erdr1* gene, ii) induces a remarkably limited suite of downstream gene expression changes developmentally, with several of relevance to associated neurobehavioural phenotypes and iii) does not elicit large changes in brain steroid biochemistry. It is possible that individuals with *STS*/*ASMT* deficiency exhibit a similarly specific pattern of gene expression changes to the 39,X^Y*^O mouse, and that these contribute towards their abnormal neurobiology. Future work may focus on whether complement pathway function, mitochondrial metabolism and cholesterol biosynthesis pathways are perturbed in such subjects.

## Background

Autism spectrum disorders (ASDs) and Attention Deficit Hyperactivity Disorder (ADHD) exhibit partially overlapping neurobehavioural symptoms, frequent comorbidity, altered monoaminergic function, and shared genetic aetiology [1]. Both types of disorder are significantly more frequently diagnosed in males than in females, suggesting a potential role for sex-linked genetic risk variants [2], and both can have long-term adverse consequences for example, increased risk for alcohol dependence in ADHD [3] or lack of independence, close social ties and employment in ASDs [4].

Both cytogenetic deletions at Xp22.32 encompassing the X-linked *STS* gene (encoding the enzyme steroid sulphatase) and its immediate neighbours, and inactivating point mutations within *STS*, appear to predispose to ADHD (particularly the inattentive subtype) [5]; larger cytogenetic deletions encompassing *STS* and more distant contiguous genes (notably *NLGN4X*) seem to predispose to autism and related disorders [5]. Polymorphisms within *STS* are associated with ADHD risk [6,7] and cognitive function in individuals with ADHD [8], whilst the gene is expressed in regions of the developing brain whose structure is known to be altered in ADHD cases [8]. Steroid sulphatase cleaves sulphate groups from a variety of steroid hormones (for example, dehydroepiandrosterone sulphate, DHEAS) thereby altering their activity and/or specificity, and subsequent developmental and physiological effects [9]. As sulphated and non-sulphated steroid hormones can act as modulators at key neurotransmitter receptors, including N-methyl-D-aspartic acid (NMDA) and γ-aminobutyric acid type A (GABA_A_) receptors [9], lack of STS developmentally could potentially elicit important effects on neuronal organisation processes mediated by these neurotransmitters [10].

Inactivating mutations within the *ASMT* gene, located within the pseudoautosomal region of the human X chromosome and encoding the enzyme acetylserotonin O-methyltransferase that catalyses the final step in melatonin biosynthesis, have been suggested as being potentially pathogenic in a variety of psychiatric and developmental conditions, including ASDs [11-17]. Such mutations may act to reduce systemic melatonin levels, a reported feature of individuals with ASDs [15]. Alternatively, or additionally, they could affect upstream substrate levels in the brain or blood platelets, for example, of the growth factor serotonin (5-hydroxytryptamine, 5-HT) or blood cell function [15]; elevated platelet serotonin levels are a consistent finding in ASD cases [18].

The 39,X^Y*^O mouse lacks both the pseudoautosomal *Sts* and *Asmt* genes (and hence their expression in all tissues) as a consequence of an end-to-end fusion of the X and Y chromosomes [19]; as such, it has some degree of construct validity as a genetic mouse model for neurodevelopmental disorders. On an MF1 outbred albino strain background, this mouse also exhibits considerable face validity for such disorders: it is inattentive [20], hyperactive, emotionally hyper-reactive (showing increased indices of stress in novel or arousing environments), occasionally aggressive [21], and perseverative (showing persistent responding in the absence of reinforcement) [19,22] and exhibits reduced systemic DHEA levels [21]. Whilst melatonin levels in wildtype and 39,X^Y*^O MF1 male mice remain to be determined, other outbred albino strains are known to produce significant quantities of the hormone [23].

Currently, the neurobiology of the 39,X^Y*^O mouse is poorly defined, although we have previously shown that it exhibits altered monoaminergic chemistry, notably elevated hippocampal and striatal serotonin levels and reduced 5-HT turnover in these regions [19,22]. Interestingly, however, the 39,X^Y*^O mouse, in contrast to individuals with ADHD, exhibits enhanced behavioural inhibition relative to 40,XY male controls as indexed by performance on murine versions of the 5-choice Serial Reaction Time Task and the Stop Signal Reaction Time Task [20, S.T., O.A.O. and W.D., unpublished observations]. We have previously shown that acute administration of one dose of the specific steroid sulphatase inhibitor COUMATE to wildtype male mice also results in inattention [20] and enhanced behavioural inhibition [S.T., O.A.O. and W.D., unpublished observations], suggesting that these phenotypes in the 39,X^Y*^O mouse are due to the ongoing activity of the enzyme. Other phenotypes in the 39,X^Y*^O mouse (for example, hyperactivity and anxiety) cannot be recapitulated by acute inhibition of steroid sulphatase [21], suggesting that they may arise from the developmental effects of deficiency for the enzyme, or alternatively from neuroendocrinological abnormalities as a consequence of ASMT deficiency.

Here, we further investigated the neurobiology of the 39,X^Y*^O mouse using two methods, with a view to identifying biological correlates of the behaviours mentioned above. First, we compared gene expression in adult 40,XY and 39,X^Y*^O whole brain tissue by microarray to identify non-obvious genetic changes between the two groups, that is changes that could not be predicted *a priori* on the basis of known biology; we assayed whole brain tissue given that *Sts* is widely expressed throughout the mouse brain, and because disruptions to multiple brain regions were likely to underpin the 39,X^Y*^O behavioural phenotypes. We also tested to see whether any of the significant changes were seen in the 39,X^Y*^O hippocampus or striatum (given the known changes in 5-HT levels), or in COUMATE-treated male mouse whole brain (and hence whether they could explain the effects of acute STS deficiency on attention and behavioural inhibition). Second, we characterised brain steroids in the adult 40,XY mouse for the first time, and compared this profile to that of the 39,X^Y*^O mouse. We anticipated that these combined approaches might reveal new pathophysiological mechanisms underlying phenotypes associated with *STS*/*ASMT* deficiency specifically and neurodevelopmental disorders more generally, and might highlight novel factors protecting against behavioural disinhibition. We report that 39,X^Y*^O mice show a highly specific pattern of gene expression changes, with several being of relevance to developmental disorders, but surprisingly, no large changes in brain steroid levels.

## Methods

### Subjects

All experimental research involving animals was performed in accordance with the Animal (Scientific Procedures) Act 1986 (United Kingdom) and was approved by the UK Home Office. 39,X^Y*^O and 40,XY mice, matched for genetic background (predominantly MF1 strain), were bred and genotyped for the presence or absence of *Sts* at the Medical Research Council National Institute for Medical Research (MRC NIMR) as described previously [22]; at six weeks of age, behaviourally naïve, group-housed 40,XY (n = 10) and 39,X^Y*^O (n = 10) mice were culled by cervical dislocation between 1200 and 1500 hours, and their brains frozen immediately at −80°C for subsequent RNA extraction. Some 40,XY and 39,X^Y*^O mice were subsequently transferred to Cardiff University, where they were treated with antibiotics to treat a *Pasteurella pneumotropica* infection prior to release onto the open racks and behavioural testing; these mice were then culled by cervical dislocation between 1100 and 1300 hours (aged 10 to 12 months), and their hippocampi and striata dissected from the left hemisphere and frozen immediately at −80°C (n = 6 per group). For the pharmacological study, behaviourally naïve, six-week old, male MF1 mice (Harlan, UK) were administered 7-O-sulphamoyl-4-methylcoumarin (COUMATE) at a dose previously shown to influence behaviour [20] (10 mg/kg in 0.5% methylcellulose 0.9% saline vehicle, per os (po), n = 12) or vehicle alone (n = 12); 24 hours later they were culled by cervical dislocation between 1600 and 1830 hours, their brains dissected and immediately frozen at −80°C. For the brain steroid measurements, behaviourally-naïve, group-housed mice (n = 8 per group) from MRC NIMR were culled by cervical dislocation between 1100 and 1500 hours (aged five to seven months), their brains dissected and frozen immediately at −80°C.

### RNA extraction and cDNA biosynthesis

High quality total RNA (absorbance ratios of 1.7 to 2.1) for the microarray study was extracted from 40,XY and 39,X^Y*^O hemibrain tissue using RNeasy Lipid Tissue Kit (Qiagen, Manchester, UK), and DNase-treated using RNase-free DNase kit (Qiagen, Manchester, UK). For other tissue samples, high quality RNA was extracted by homogenising the tissue in TRIReagent (Sigma-Aldrich, Gillingham, UK), precipitating with isopropanol, washing in 75% ethanol, and resuspending in RNase-free distilled water; DNase treatment was then carried out using the TURBO DNA-free kit (Life Technologies, Paisley, UK). cDNA was synthesised from 1 to 2 μg RNA per sample using EcoDry Premix with random hexamers (Clontech, Mountain View, CA, USA), and diluted 25-fold.

### Microarray analysis

Total RNA was supplied to Source Bioscience Ltd. (Nottingham, UK) where it underwent quality control checks prior to conversion to cRNA and hybridisation to the Mouse Gene 1.0 ST array (Affymetrix, Santa Clara, USA) according to their standard protocol. To reduce the possibility of spurious expression differences, we carried out four separate hybridisations: 40,XY1 (five pooled hemibrains), 40,XY2 (five pooled hemibrains), 39,X^Y*^O1 (five pooled hemibrains), and 39,X^Y*^O2 (five pooled hemibrains). Array data were subject to normalisation using the Robust Multipoint Average (RMA) procedure (background adjustment, quantile normalisation and summarisation) within the Affymetrix Expression Console prior to being loaded in the CLC Bio Genomics Workbench to examine differential expression across samples. Microarray data are available via ArrayExpress [24] (accession number E-MEXP-3943).

### Quantitative polymerase chain (qPCR) analysis

Quantitative PCR (qPCR) analysis of gene expression was performed using a Rotorgene 6000 coupled with a CAS1200 automated set-up, and utilizing standard consumables (Qiagen, Manchester, UK). PCR reactions were performed using 5 μl cDNA mix and 200 nM custom-designed primers (Additional file 1) and Quantace SensiMix (Bioline, London, UK). qPCR data were analysed using ΔC_t_ methods as described previously [25]. Briefly, individual PCR reaction data were normalised to the mean of at least three ‘housekeeping gene standards’ (*Hprt*, *Gapdh*, *Actb* and *Rn18s*, whose expression was significantly correlated within the samples), giving a value known as Δ*C*_t_. The data were processed further to show how the 39,X^Y*^O values vary with respect to those of the 40,XY (ΔΔ*C*_t_) and allow simpler graphical representation after transformation (2^−ΔΔ*C*t^).

### Brain steroid analysis

Pooled samples (40,XY: n = 4, 39,X^Y*^O: n = 4) were generated for each group (two whole brains per pool). Free steroids and their sulphate esters were extracted from brain tissue and fractionated prior to derivatisation with methoxyamine (MO) and trimethylsilylimidazole (TMSI) for gas chromatography-mass spectrometry (GC-MS) as previously described [26] except that steroid sulphates were deconjugated by incubation with Helix sulphatase rather than by solvolysis [27]. Briefly, after drying down and redissolving in sodium acetate (0.5 M, pH 5.0), the sulphates were deconjugated by incubation with the arylsulphatase enzyme from *Helix pomatia* Type H1 (Sigma-Aldrich, Gillingham, UK) at 1 mg/ml overnight at 40°C, then at 55°C for two hours. The deconjugated steroids could then be recovered by loading the incubate onto a 60 mg hydrophobic-lipophilic balance (HLB) cartridge (Waters, Milford, MA, USA). After a wash with 5 ml water, the steroids were eluted from this cartridge with 4 ml ethyl acetate. These steroids liberated by deconjugation of the sulphate fraction were then dried down under nitrogen and derivatised with MO and TMSI for GC-MS alongside the steroids from the free steroid fraction of mouse brain. Increasing amounts (1.4 to 23.1 ng) of standard reference steroids were derivatised at the same time for calibration and each sample and calibration reference included a fixed amount (50 ng) of the internal standards 16-dehydropregnenolone and 6α-methyl-17-hydroxyprogesterone. Column conditions for the GC were as previously described, with elution of the MO-TMS-steroids detected by selective ion monitoring as follows: androsterone, 270 and 360; dehydroepiandrosterone, 268 and 358; epiandrosterone, 270 and 360; androstenediol, 129 and 239; 17β-oestradiol, 129, 285 and 416; testosterone, 129 and 389; allopregnanolone, 298 and 388; 16-dehydropregnenolone, 400 and 415; allopregnanediol, 269 and 346; pregnenolone, 288 and 386; epiallopregnanolone, 243 and 388; 5α-dihydroprogesterone, 288 and 343; 20β-dihydropregnenolone, 372 and 462; 20α-dihydropregnenolone, 372 and 462; progesterone, 341 and 372; 5α,20α-tetrahydroprogesterone, 289 and 303; 6α-methyl-17-hydroxyprogesterone, 443 and 474.

### Statistics

For the microarray data, statistical significance of the changes in mean expression levels per group was examined by the unpaired two-tailed *t*-test with a single overall factor of KARYOTYPE (that is 40,XY or 39,X^Y*^O) within CLC Bio Genomics Workbench; *P*-values taking into account correction for False Discovery Rate were also calculated. For the qPCR analyses, samples > two standard deviations from the group mean were excluded as outliers; statistical significance of the changes in expression levels was examined using 2^-∆Ct^ values by the unpaired *t*-test (normally distributed data according to Shapiro-Wilks test) or Mann-Whitney *U*-test (non-normally distributed data according to Shapiro-Wilks test) with a single factor of KARYOTYPE. Where variance within the two groups was unequal, Greenhouse-Geisser corrected degrees of freedom are indicated. Where the direction of effects could be predicted, that is in confirming microarray findings, a one-tailed test was used; in other cases, a two-tailed test was used. For the brain steroid hormone analyses, data were tested for normality as above, and analysed by the unpaired *t*-test with a single factor of KARYOTYPE. *P*-values ≤ 0.05 were regarded as significant, and all data are presented as mean values ± standard error of the mean.

## Results

### Microarray analysis

Filtering for nominal *P*-values < 0.05, and >2-fold change identified just 13 genes of interest in addition to *Sts*, which was, as expected, found to be significantly downregulated; of these genes, eight appeared to be upregulated, and five downregulated (Table 1). *Asmt* was not represented on the array. Just one gene, *Erdr1* (NM_133362.1) survived correction for False Discovery Rate (*P* = 0.044). Of particular note was the finding that two genes that lie adjacent to one another on chromosome 11 (*Sfi1* and *Pisd-ps1*) were both upregulated, a result highly unlikely to occur by chance. A further 1,536 transcripts (out of 26,166 RefSeq transcripts represented on the array) were potentially more subtly differentially expressed (that is *P* < 0.05, but <2-fold expression change); of these, 47 had previously been implicated in autism and/or ADHD according to relevant online databases [28-30]: *Abat*, *Arhgap15*, *Cdh8*, *Chrna7*, *Cldn5*, *Cntn3*, *Crygc*, *Cttnbp2*, *Ctnna2*, *Dctn5*, *Dhcr7*, *Dhrs3*, *Ehmt1*, *Elovl6*, *Eif4e1b*, *Eln*, *Fbxo40*, *Gclc*, *Gdi1*, *Gfod1*, *Gpd2*, *Grid2*, *Hepacam*, *Hes1*, *Hes6*, *Irak1*, *Kcnj10*, *Lphn3*, *Lrp2*, *Lrrn3*, *Mcph1*, *Mctp1*, *Mid1*, *Nlgn1*, *Npy5r*, *Nucb1*, *Park2*, *Pdzd4*, *Plcb1*, *Prodh*, *Prrt2*, *Prune2*, *Scn2a1*, *Slc25a12*, *Srsf3*, *Syngap1* and *Ythdc2*.

**Table 1:** **Genes showing >2fold change at *P*** **< 0.05 in 39,X**^Y*^O whole brain based on the microarray analysis

**Upregulated in 39,X**^Y*^O brain					
**Gene**	**Chromosome**	**Product**	**Fold change**	***P*-value**	**Human orthologue**
*Gm16432*	1	Function unknown	5.5	0.006	None
*Vmn2r86*	10	Vomeronasal 2 receptor 86	3.6	0.03	None
*Sfi1*	11	Sfi1 homolog, spindle assembly associated (yeast)	3.3	0.007	*SFI1* (22q12.2)
*Pisd-ps1*	11	Phosphatidylserine decarboxylase, pseudogene 1	2.7	0.02	None
*Tagap1*	17	T cell activation GTPase activating protein 1	2.4	0.01	None
*C1qc*	4	Complement component 1, q subcomponent, C chain	2.3	0.002	*C1QC* (1p36.11)
*Fam177a*	12	Family with sequence similarity 177, member A	2.2	0.004	*FAM177A1* (14q13.2)
*Metap1d*	2	Methionyl aminopeptidase type 1D	2.0	0.0003	*METAP1D* (2q31.1)
**Downregulated in 39,X**^Y*^O brain					
**Gene**	**Chromosome**	**Product**	**Fold change**	**p-value**	**Human orthologue**
*Erdr1*	Y	Erythroid differentiation regulator 1	3.6	1.3x10^−6^	None
*Trdn*	10	Triadin	3.1	0.03	*TRDN* (6q22.31)
*Gm12696*	4	Pseudogenic RNA	2.9	0.01	None
*Fut11*	14	Fucosyltransferase 11	2.2	0.002	*FUT11* (10q22.2)
*AA388235*	17	Non-coding RNA	2.1	0.03	None

### Confirmation of microarray results by quantitative PCR (qPCR)

**Figure 1:**
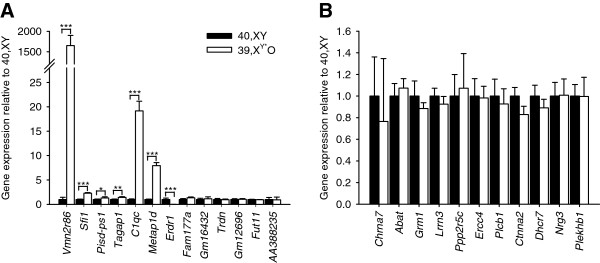
**Gene expression in 40,XY and 39,X**^Y*^O adult hemibrain tissue. Of the thirteen microarray calls showing >2-fold change with *P*-values < 0.05, seven were confirmed by quantitative PCR **(A)**. Of 11 genes previously implicated in neurodevelopmental phenotypes, and having microarray calls with *P*-values < 0.05, none were found to be significantly differentially expressed between the two experimental groups **(B)**. *P < 0.05, **P < 0.01, ***P < 0.001.

Initially, we sought to confirm microarray changes > 2-fold and with a *P*-value < 0.05 by qPCR using individual samples (n = 10 per group). The direction of effect was consistent between the microarray and the qPCR analysis for 12 of the 13 genes in Table 1. However, the expression of only seven of the 13 genes was confirmed as being significantly different between the two experimental groups by qPCR (Figure 1A): six upregulated: *Vmn2r86*: t_8.00_ = −6.11, *P* < 0.001 (expression higher than 40,XY mean in 10/10 39,X^Y*^O samples), *Sfi1*: t_16_ = −8.62, *P* < 0.001 (expression higher than 40,XY mean in 10/10 39,X^Y*^O samples), *Pisd-ps1*: t_11.84_ = −1.84, *P* < 0.05 (expression higher than 40,XY mean in 7/10 39,X^Y*^O samples), *Tagap1*: t_17_ = −3.32, *P* < 0.005 (expression higher than 40,XY mean in 8/10 39,X^Y*^O samples), *C1qc*: t_9.07_ = −11.47, *P* < 0.001 (expression higher than 40,XY mean in 10/10 39,X^Y*^O samples), *Metap1d*: t_9.29_ = −12.02, *P* < 0.001 (expression higher than 40,XY mean in 10/10 39,X^Y*^O samples), and one downregulated: *Erdr1*: t_9.00_ = 5.96, *P* < 0.001 (expression lower than 40,XY mean in 10/10 39,X^Y*^O samples). *Prima facie*, these genes do not appear to function within common known pathways, being involved in signal transduction (*Vmn2r86*), phosphatase activity regulation (*Sfi1*), complement activation in the immune response (*C1qc*), mitochondrial proteolysis (*Metap1d*) and regulation of cell proliferation and migration (*Erdr1*) [31]. The expression of *Fam177a* showed a trend towards being more highly expressed in 39,X^Y*^O brain than 40,XY brain (t_17_ = −1.51, *P* = 0.08). The expression of the *Gm16432*, *Trdn*, *Gm12696*, *Fut11* and *AA388235* genes did not differ significantly as a function of KARYOTYPE (t_18_ = −0.67, *P* = 0.26, t_18_ = 0.45, *P* = 0.33, t_18_ = −0.18, *P* = 0.43, t_17_ = 1.08, *P* = 0.15 and *P* = 0.94 respectively).

The *Erdr1* gene is located adjacent to the fusion point of the X and Y chromosomes in the 39,X^Y*^O mouse; therefore we tested whether two additional genes not represented on the microarray, which lie directly adjacent to *Erdr1* (*G530011O06Rik* and *LOC100861696*) [19] were also differentially expressed in 39,X^Y*^O and 40,XY brain; this did not appear to be the case: *G530011O06Rik*: t_13.4_ = −0.037, *P* = 0.97, *LOC100861696*: *P* = 0.33 (see Additional file 2).

Subsequently, we examined the expression of a selection of genes with known, or suspected, roles in neurodevelopmental disorders with < 2-fold change (1.04 to 1.25-fold change) but *P*-values < 0.05 using qPCR. Of the 11 genes examined, none were confirmed as being significantly differentially expressed between 40,XY and 39,X^Y*^O groups (Figure 1B, *Chrna7*: t_17_ = −0.15, *P* = 0.44, *Abat*: *P* = 0.33, *Grm1*: t_17_ = 1.26, *P* = 0.12, *Lrrn3*: t_18_ = 0.77, *P* = 0.23, *Ppp2r5c*: t_17_ = −0.62, *P* = 0.28, *Ercc4*: t_18_ = 0.32, *P* = 0.38, *Plcb1*: t_16_ = 0.41, *P* = 0.34, *Ctnna2*: t_17_ = 1.71, *P* = 0.06, *Dhcr7*: t_18_ = 1.04, *P* = 0.16, *Nrg3*: t_18_ = −0.15, *P* = 0.45, *Plekhb1* t_18_ = −0.27, *P* = 0.40).

### Gene expression changes in the hippocampus and striatum of 39,X^Y*^O mice

**Figure 2:**
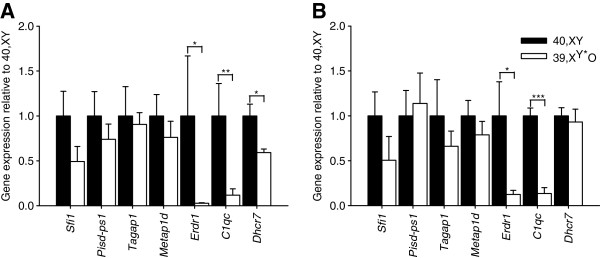
**Gene expression in 40,XY and 39,X**^Y*^O adult brain regions. Of six candidate genes confirmed as being differentially expressed by microarray and known to be expressed in the hippocampus, two were significantly downregulated in this brain region in 39,X^Y*^O mice (*Erdr1* and *C1qc*); a third gene, *Dhcr7*, implicated in serotonergic neurodevelopmental phenotypes, was also downregulated in mutant mice **(A)**. *Erdr1* and *C1qc* were also significantly downregulated in the 39,X^Y*^O striatum **(B)**.*P < 0.05, **P < 0.01, ***P < 0.001.

Of the seven genes whose expression levels differed in 40,XY and 39,X^Y*^O whole brain tissue, six were expressed in the adult hippocampus, and all showed reduced mean levels in 39,X^Y*^O tissue (Figure 2A). However, for only two of these (*Erdr1* and *C1qc*), was this reduction statistically significant: *Sfi1*: t_10_ = 1.81, *P* = 0.10, *Pisd-ps1*: t_10_ = 1.20, *P* = 0.26, *Tagap1*: t_10_ = 0.78, *P* = 0.45, *Metap1d*: t_10_ = 1.04, *P* = 0.33, *Erdr1*: t_5.00_ = 3.17, *P* = 0.025 (expression lower than 40,XY mean in 6/6 39,X^Y*^O samples), *C1qc*: *P* = 0.004 (expression lower than 40,XY mean in 6/6 39,X^Y*^O samples)). We also examined hippocampal expression of *Dhcr7* (encoding 7-dehydrocholesterol reductase, *P* < 0.05 in the microarray analysis) given that this gene had previously been implicated in neurodevelopmental disorders, and that its deficiency had been implicated in altered serotonergic function [32]. We found that in 39,X^Y*^O hippocampus, *Dhcr7* expression was significantly downregulated relative to 40,XY expression: t_5.97_ = 3.47, *P* = 0.013 (expression lower than 40,XY mean in 6/6 39,X^Y*^O samples) (Figure 2A).

Of the seven genes whose expression levels differed in 40,XY and 39,X^Y*^O whole brain tissue, six were expressed in the adult striatum; we also examined *Dhcr7* expression in this tissue. Both *Erdr1* and *C1qc* genes were found to be significantly downregulated in 39,X^Y*^O striatum: t_5.19_ = 3.12, *P* = 0.025 and t_10_ = 8.69, *P* < 0.001 respectively (expression lower than 40,XY mean in 6/6 39,X^Y*^O samples for both genes). The expression of the remaining five genes was equivalent in 40,XY and 39,X^Y*^O striatum (Figure 2B, *Sfi1*: t_10_ = 1.35, *P* = 0.21, *Pisd-ps1*: t_10_ = −0.58, *P* = 0.58, *Tagap1*: t_6.59_ = 1.29, *P* = 0.24, *Metap1d*: t_10_ = 1.04, *P* = 0.32, *Dhcr7*: t_10_ = 0.33, *P* = 0.75).

### Brain gene expression changes in wildtype COUMATE-treated male mice

To determine whether the behavioural effects arising from acute steroid sulphatase inhibition could be due to altered expression of the *Vmn2r86*, *Sfi1*, *Pisd-ps1*, *Tagap1*, *C1qc*, or *Map1d* genes, we compared their expression in COUMATE and vehicle-treated MF1 male hemibrain. *Erdr1* expression was not examined in COUMATE-treated brain given that its reduced expression in the 39,X^Y*^O brain was due to a direct effect of the genetic mutation and not a downstream effect of *Sts* or *Asmt* deficiency. None of the six genes assayed were significantly differentially expressed in these tissues: *Vmn2r86* (t_22_ = −0.99, *P* = 0.33), *Sfi1* (t_21_ = 0.01, *P* = 0.99), *Pisd-ps1* (t_20_ = 0.84, *P* = 0.41), *Tagap1* (t_22_ = −0.82, *P* = 0.42), *C1qc* (t_22_ = −0.42, *P* = 0.68), *Metap1d* (t_21_ = 0.64, *P* = 0.53) (Additional file 3).

### Steroid hormone analysis

Of the 15 steroids whose identity could reliably be determined from their elution and ion profile in our GC-MS analyses, ten had concentrations above the limit of detection in the free steroid fraction: (dehydroepiandrosterone (DHEA), epiandrosterone, testosterone, allopregnanolone, pregnenolone, epiallopregnanolone, 5α-dihydroprogesterone, 20α-dihydropregnenolone, progesterone and 5α,20α-tetrahydroprogesterone). The concentrations of these ten steroids did not differ significantly between 40,XY and 39,X^Y*^O brains, although there was considerable intra-group variability, particularly in the mutant group (Table 2).

**Table 2:** **Levels of free and sulphated steroids in whole adult 40,XY and 39,X**^Y*^O mouse brain (n = 4 per group)

**Steroid (systematic name in brackets)**	**Free steroids**			**Steroid sulphates**		
	**40,XY Concentration (ng/g)**	**39,X**^Y*^O Concentration (ng/g)	**Significance t**_**6**_	**40,XY Concentration (ng/g)**	**39,X**^Y*^O Concentration (ng/g)	**Significance t**_**6**_
Androsterone (5α-androstan-3α-ol-17-one)	ND; < 4.56	ND; < 4.56	N/A	ND; < 0.63	ND; < 0.63	N/A
dehydroepiandrosterone (5-androsten-3β-ol-17-one)	36.15 ± 9.74	32.91 ± 15.21	0.18, *P* = 0.86	0.51 ± 0.02	0.53 ± 0.01	−1.34, *P* = 0.23
Epiandrosterone (5α-androstan-3β-ol-17-one)	2.80 ± 0.12	3.60 ± 0.44	−1.77, *P* = 0.13	0.71 ± 0.02	0.72 ± 0.01	−0.66, *P* = 0.54
Androstenediol (5-androsten-3β,17β-diol)	ND; < 0.01	ND; < 0.01	N/A	ND; < 0.45	ND; < 0.45	N/A
17β-oestradiol(1,3,5(10)-oestratrien-3,17β-diol)	ND; < 0.53	ND; < 0.53	N/A	ND; < 0.05	ND; < 0.05	N/A
Testosterone (4-androsten-17β-ol-3-one)	19.64 ± 2.27	19.63 ± 9.81	0.00, *P* = 1.00	ND; < 1.77	ND; < 1.77	N/A
Allopregnanolone (5α-pregnan-3α-ol-20-one)	1.47 ± 0.27	1.95 ± 0.77	−0.59, *P* = 0.58	ND; < 0.01	ND; < 0.01	N/A
Allopregnanediol (5α-pregnan-3α,20α-diol)	ND; < 0.01	ND; < 0.01	N/A	ND; < 0.01	ND; < 0.01	N/A
Pregnenolone (5-pregnen-3β-ol-20-one)	31.26 ± 13.37	25.12 ± 15.05	0.31, *P* = 0.77	ND; < 0.01	ND; < 0.01	N/A
Epiallopregnanolone (5α-pregnan-3β-ol-20-one)	17.07 ± 5.13	25.25 ± 17.19	−0.46, *P* = 0.67	ND; < 0.37	ND; < 0.37	N/A
5α-dihydroprogesterone (5α-pregnan-3,20-dione)	12.75 ± 5.26	15.01 ± 6.16	−0.28, *P* = 0.79	ND; < 0.01	ND; < 0.01	N/A
20β-dihydropregnenolone (5-pregnen-3β,20β-diol)	ND; < 3.10	ND; < 3.10	N/A	ND; < 0.01	ND; < 0.01	N/A
20α-dihydropregnenolone (5-pregnen-3β,20α-diol)	0.91 ± 0.11	0.94 ± 0.09	−0.21, *P* = 0.84	ND; < 0.01	ND; < 0.01	N/A
Progesterone (4-pregnen-3,20-dione)	3.74 ± 0.34	5.84 ± 2.05	−1.01, *P* = 0.35	ND; < 4.41	ND; < 4.41	N/A
5α,20α-tetrahydroprogesterone (5α-pregnan-20α-ol-3-one)	3.71 ± 0.22	3.97 ± 0.48	−0.50, *P* = 0.64	ND; < 1.61	ND; < 1.61	N/A

N/A not applicable, ND not detectable.

In the steroid sulphate fraction, two steroids had concentrations above the limit of detection (DHEAS and epiandrosterone sulphate); the concentrations of both of these steroids did not differ significantly in 40,XY and 39,X^Y*^O brain (Table 2).

## Discussion

The 39,X^Y*^O mouse model exhibits face and construct validity for neurodevelopmental disorders, showing behavioural endophenotypes associated with such conditions, and lacking two genes (*Sts* and *Asmt*) whose human orthologues have been implicated in ADHD and autism pathogenesis respectively. In this study, we investigated the neurobiology of this model by two methods with a view to identifying mechanisms by which loss of function of these genes might contribute towards behavioural pathology; this work is important given the current lack of availability of single gene knockout models for *Sts* and *Asmt*.

Microarray and quantitative PCR analyses comparing 40,XY and 39,X^Y*^O brain tissue identified a surprisingly small number of robust gene expression differences between the groups. Our inability to verify differential microarray expression calls at *P* < 0.05 for multiple transcripts of relevance to autism and ADHD suggests that we are likely to have successfully identified all the genes that truly differ in their expression across the whole brain in 40,XY and 39,X^Y*^O mice. However, it is possible that there are further group differences in gene expression within specific brain regions; indeed, our microarray analysis did not indicate altered expression of *Htr2c* (encoding the serotonin 2c receptor) which we have previously shown to be upregulated in the 39,X^Y*^O hippocampus [22].

*Erdr1* gene expression was significantly reduced in 39,X^Y*^O whole brain, and in hippocampal and striatal dissections; this gene is retained in the 39,X^Y*^O mouse and is located adjacent to the fusion point of the X and Y chromosomes [19]. Our current results suggest that in addition to deleting of the *Sts* and *Asmt* genes, the lesion in 39,X^Y*^O mice disrupts a genetic element that enhances *Erdr1* expression. Theoretically, in concert with loss of *Sts* and *Asmt*, reduced expression of the widely-expressed, but poorly-characterised, erythroid differentiation regulator 1 protein encoded by *Erdr1* could contribute towards downstream gene expression changes, abnormal striatal and hippocampal monoamine neurochemistry, and behavioural phenotypes in the 39,X^Y*^O model; brain and behavioural investigations in mice in which the function of this gene alone is disrupted will help clarify the extent and specificity of this contribution.

Our microarray and qPCR analyses also showed that the expression of *C1qc* (encoding the protein complement component 1, q subcomponent, c chain) was upregulated in the brains of young, behaviourally-naïve 39,X^Y*^O mice, but downregulated in the striata and hippocampi of older, behaviourally-trained mutant animals. These data suggest a potential basis for the neurochemical and behavioural abnormalities seen in the 39,X^Y*^O mouse that may be sensitive to spatiotemporal or environmental regulation, and further suggest the possibility that the neurobehavioural pathology in individuals lacking functional STS and/or ASMT proteins may be due, in part, to altered C1QC levels. Previous animal and clinical studies have implicated aberrant expression of C1q family members in developmental and behavioural phenotypes. In rodents, C1q deletion results in altered synaptic elimination [33,34], *C1qc* expression levels are altered in a model of developmental hippocampal pathology [35], and *C1qc* expression is associated with behavioural phenotypes (notably the consumption of ethanol relative to water) [36]. In man, individuals with autism can exhibit elevated C1QC serum levels [37] and altered gastrointestinal C1q deposition [38,39]. Whether the 39,X^Y*^O mouse exhibits alterations in synaptic structure/function or hippocampal structure, or heightened alcohol preference remains to be investigated. It also remains to be seen whether individuals with elevated C1QC levels and autism possess genetic mutations in either *STS* or *ASMT*, and whether individuals lacking functional *STS* and/or *ASMT* genes are at increased risk of alcohol dependence.

By the same logic, the present findings further indicate that disrupted expression of *Metap1d* and/or *Sfi1* could play a role in 39,X^Y*^O phenotypes, and in developmental phenotypes associated with Xp22.3 mutations, but probably not through influencing striatal or hippocampal physiology. There is some evidence for an association between a linkage block at 2q31.1 containing *METAP1D* and autism [40], whilst copy number variants encompassing *SFI1* have previously been identified in autism and related developmental disorders [41-45].

We also showed that the genetic mutation in 39,X^Y*^O mice resulted in reduced hippocampal expression of the *Dhcr7* gene. DHCR7 is a known modulator of serotonergic system development in mammals [32]; therefore, its reduced expression represents a strong candidate mechanism for abnormal serotonin levels in the 39,X^Y*^O hippocampus and associated behavioural phenotypes [19,22]. In man, defects in the DHCR7 enzyme underlie Smith-Lemli-Opitz syndrome (SLOS). Individuals with SLOS exhibit a range of behavioural symptoms with some overlap with autism, including: hyperactivity, aggression, insomnia, self-injurious behaviour, sensory hypersensitivity and repetitive behaviours [32]; interestingly, several of these behavioural abnormalities are also observed in the 39,X^Y*^O mouse [19,21,22], indicating that hippocampal loss of DHCR7 function may underlie key SLOS phenotypes, and suggesting the 39,X^Y*^O mouse as a potential novel model for aspects of the syndrome.

Genetic and functional work in mice has indicated a link between steroid sulphatase and aggressive behaviour [46,47]. Consistent with this, 39,X^Y*^O mice [21], and mice co-administered COUMATE and DHEAS [48], exhibit elevated levels of aggression. Previous rodent studies have demonstrated that major urinary proteins may elicit aggressive behaviour through their actions at sensory neurons expressing Vmn2r putative pheromone receptors [49]. Of the 124 genes within the *Vmn2r* family, the expression of just one, *Vmn2r86*, was significantly altered in 39,X^Y*^O brain; this increased expression thus represents an excellent candidate mechanism underlying aggression in these mutant mice. The fact that there is no human orthologue of *Vmn2r86* may explain why individuals with Xp22.3 deletions encompassing *STS* and/or *ASMT* do not consistently show obvious aggressive tendencies.

Acute administration of COUMATE, a specific steroid sulphatase inhibitor, given at a dose known to induce behavioural changes, did not recapitulate any of the whole brain gene expression changes seen in the 39,X^Y*^O mouse. There are two obvious possibilities why this might be the case: i) the expression changes in the 39,X^Y*^O mouse result from abnormal developmental expression of STS and/or ii) the gene expression changes in the 39,X^Y*^O mouse are the result of loss of function of ASMT, or reduced expression of *Erdr1*. A previous study found no effect of acute administration of COUMATE on the concentrations of endogenous DHEAS or DHEA in whole mouse brain, although the drug did reduce entry of systemic DHEAS into the brain [50]. Thus, the molecular basis of COUMATE-induced behavioural changes remains obscure; it is plausible that the drug induces brain region-specific gene expression changes that we were unable to detect, and this possibility remains to be investigated.

Our current analyses provide, for the first time, a systematic profile of the steroid milieu in the mouse brain. There was substantial overlap between the free and sulphated steroids that were detectable in the adult male mouse brain (present data), and those that were most readily detectable in the adult male rat brain [26] consistent with a degree of cross-species homology, although, interestingly, concentrations of most compounds tended to be higher in mouse brain. We found no significant differences in the concentrations of the detectable compounds between 40,XY and 39,X^Y*^O brains, consistent with an absence of large between-group differences in steroid brain biochemistry. This finding, taken together with our previous data showing reduced serum DHEA levels in 39,X^Y*^O mice [19] suggests the possibility that where STS is absent developmentally, as in 39,X^Y*^O mice, a compensatory mechanism is recruited to cleave sulphated steroid esters in brain, but not in peripheral tissues. Due to the difficulty of generating 39,X^Y*^O mice and precisely genetically-matched controls, and the apparent variability in brain steroid levels in the mutant group, our study had limited power, with several steroids below the limit of detection. As such, we cannot completely exclude the possibility that there are subtle differences in levels of one or more steroids within 40,XY and 39,X^Y*^O brain tissue.

## Conclusions

Here, we have shown that the genetic mutation in 39,X^Y*^O mice, in addition to deleting the *Sts* and *Asmt* genes, also results in significant downregulation of the adjacent *Erdr1* gene. In the absence of *Sts* or *Asmt* single gene knockout mice, the present study gives the first clues as to possible downstream gene expression changes that might result from the loss of one (or both) of these genes; of the limited number of robust resultant gene expression changes, several may be pertinent to 39,X^Y*^O neurochemical and behavioural phenotypes, and hence, to similar phenotypes in individuals with loss of function mutations within *STS* or *ASMT*. Importantly, it should be noted that just because gene expression differences across genotypes are large, they might not necessarily be biologically significant.

Future functional validation and pathway analysis studies in the 39,X^Y*^O mouse throughout development, incorporating an examination of the spatiotemporal dynamics of protein changes indicated by our present study, should further elucidate the neurobiological pathways by which the 39,X^Y*^O mutation gives rise to behavioural phenotypes analogous to those seen in neurodevelopmental disorders. Future work might examine whether the gene expression changes seen here are recapitulated in accessible tissues from individuals lacking STS and/or ASMT, and could test for abnormalities in complement pathway function, mitochondrial metabolism and cholesterol biosynthesis. Should this be the case, these physiological abnormalities could modulate either the risk of developing ADHD or autism, and/or to the clinical course of these disorders.

Our analyses indicated no large differences in brain steroid concentration between 40,XY and 39,X^Y*^O adult mice, and hence, suggest that altered steroid biochemistry may not be a significant contributor to abnormal brain and behavioural phenotypes in this mouse model, nor to similar phenotypes in individuals with mutations in *STS* and/or *ASMT*. However, a developmental difference in brain steroid levels between *Sts*/*Asmt*-deficient and wildtype subjects may plausibly exist and contribute towards between-group adult behavioural phenotypes.

## Abbreviations

ADHD: Attention Deficit Hyperactivity Disorder; ASDs: autism spectrum disorders; DHEA(S): dehydroepiandrosterone (sulphate); GABA: γ-aminobutyric acid; GC-MS: gas chromatography-mass spectrometry; HLB: hydrophobic-lipophilic balance; 5-HT: 5-hydroxytryptamine; MO: methoxyamine; MRC NIMR: Medical Research Council National Institute for Medical Research; NMDA: N-acetyl-D-aspartic acid; PCR: polymerase chain reaction; po: per os; qPCR: quantitative polymerase chain reaction; RMA: Robust Multipoint Average; SLOS: Smith-Lemli-Opitz syndrome; STS: steroid sulphatase; TMSI: trimethylsilylimidazole.

## Competing interests

The authors declare that they have no competing interests.

## Authors' contributions

ST, JPF and WD conceived and designed the experiments. ST, JPF and OAO performed the experiments. ST, JPF and WD analysed the data. ST, JFP, OAO and WD wrote the paper. All authors read and approved the final manuscript.

## Supplementary Material

Additional file 1 Primer sequences for quantitative PCR analyses. Click here for file

Additional file 2 **Expression of genes adjacent to *Erdr1* in 40,XY and 39,X**^Y*^O hemibrain tissue. Click here for file

Additional file 3 Comparison of gene expression in vehicle and COUMATE-treated 40,XY mouse brain. Click here for file
