# Supplementary material for: Altered brain gene expression but not steroid biochemistry in a genetic mouse model of neurodevelopmental disorder
Source: Mol Autism. 2014 Mar 6;5:21. doi: 10.1186/2040-2392-5-21 (PMC3946266; doi:10.1186/2040-2392-5-21)
Supplement: Additional file 1 — Primer sequences for quantitative PCR analyses. [file 2040-2392-5-21-S1.docx]

**Trent et al. Altered brain gene expression but not steroid biochemistry in a genetic mouse model of neurodevelopmental disorder**

**Additional file 1**

Primer sequences for quantitative PCR analyses

| **Gene** | **Forward primer (5’-3’)** | **Reverse primer (5’-3’)** |
| --- | --- | --- |
| *Gapdh* | GAACATCATCCCTGCATCCA | CCAGTGAGCTTCCCGTTCA |
| *Hprt* | TTGCTCGAGATGTCATGAAGGA | AATGTAATCCAGCAGGTCAGCAA |
| *Actb* | TCTGTGTGGATTGGTGGCTCTA | CTGCTTGCTGATCCACATCTG |
| *Rn18s* | GTAACCCGTTGAACCCCATT | CCATCCAATCGGTAGTAGCG |
| *Vmn2r86* | CTCCTCCATTTGCAGTGTGA | ACCTCATTTTCTGGGCACTG |
| *Sfi1* | GCCATCTAGAAGCTGCCACT | GAACTTTCTGGCCACACACC |
| *Pisd-ps1* | ACGAGTTTGCTGTCATGTGC | TCAGTCATGTTCACCCCAAA |
| *Tagap1* | CACCAAATTTTGCCTGTGTG | CATGCATGAAGGGATGTACG |
| *C1qc* | CAGCGTCTTCTCTGGTTTCC | TCCTGGAGGAAGAGGTCTGA |
| *Metap1d* | GTTAGTGGAGGTTGCCAGGA | CTGATGAGTTATGCGGCTGA |
| *Erdr1* | CTTTTAGCCGCAGCTATGGT | ATTCACGCCCACAGAGAAAC |
| *Fam177a* | AACATGGGTGGGTGAACATT | TAACCCGGGATGACTGATGT |
| *Gm16432* | GGCCTGGAGACAGCTTAGTG | CTGGGGCCATTCAAAATAAA |
| *Trdn* | AGAAGCAAACACGTCCCATC | CCAGGTTGTTGGACAGGAGT |
| *Gm12696* | ACGGGGATCTTCTTCCAAGT | TCCAGGCTTTCCAGAAGAGA |
| *Fut11* | GGACAGGATGGTTCAGGCTA | ACCCACACATAAGGCTTTGC |
| *AA388235* | GCTCTCTCGCTGCCTAAAGA | TTCTGCGGCAAAGCTTTATT |
| *Chrna7* | CCGGAGTGAAAAATGTTCGT | CAAGACGTTGGTGTGGAATG |
| *Abat* | GGCCACTGCTAGACTTCCTG | ACCATCAGGAACCAACAAGC |
| *Grm1* | GGAAGTGTGGGGAGATCAGA | TCTCACTGCCCAGAGTGATG |
| *Lrrn3* | AGAGTCTTTGCCGAACCTCA | TCCACACAGAACAGCGAGTC |
| *Ppp2r5c* | CCCAGAAGAGGATGAACCAA | TGGGTTGGAAATCTGGAGAC |
| *Ercc4* | AAGTGTCCCCCACTTCACTG | GGAGAAGCAGTCAGGACAGG |
| *Plcb1* | CAGAAGTAGAGGCGCAAACC | CTCCGTGGTTTTCTTGTGGT |
| *Ctnna2* | GATGTGGACACACTGGATCG | GTGGCTTCCAGCACTTTCTC |
| *Dhcr7* | AGACATTTGGGCCAAGACAC | AAGCCAGGAATAAAGCAGCA |
| *Nrg3* | GCATCCAGCACAAAGTCTGA | CTGGGGAGCTGAAAAACTTG |
| *Plekhb1* | TTCTCCCCATTAAGCCCTCT | TGGCTATGTCTGTGCCTGAG |
| *G530011O06Rik* | TTGTGGCATTGCACTTTCAT | TCACTCCGCTGCAGACATAC |
| *LOC100861696* | ACAGGGAGGAGACACAGAGC | ATCCCAGGAGCGAGTGTG |
