# Supplementary material for: Altered brain gene expression but not steroid biochemistry in a genetic mouse model of neurodevelopmental disorder
Source: Mol Autism. 2014 Mar 6;5:21. doi: 10.1186/2040-2392-5-21 (PMC3946266; doi:10.1186/2040-2392-5-21)
Supplement: Additional file 2 — Expression of genes adjacent to Erdr1 in 40,XY and 39,X Y* O hemibrain tissue. [file 2040-2392-5-21-S2.docx]

**Trent *et al.* Altered brain gene expression but not steroid biochemistry in a genetic mouse model of neurodevelopmental disorder**

**Additional File 2**

Expression of two genes adjacent to *Erdr1* in 40,XY and 39,X^Y*^O hemibrain tissue (n=10 per group)
