# Supplementary material for: Altered brain gene expression but not steroid biochemistry in a genetic mouse model of neurodevelopmental disorder
Source: Mol Autism. 2014 Mar 6;5:21. doi: 10.1186/2040-2392-5-21 (PMC3946266; doi:10.1186/2040-2392-5-21)
Supplement: Additional file 3 — Comparison of gene expression in vehicle and COUMATE-treated 40,XY mouse brain. [file 2040-2392-5-21-S3.docx]

**Trent et al. Altered brain gene expression but not steroid biochemistry in a genetic mouse model of neurodevelopmental disorder**

**Additional file 3**

**Brain gene expression in wildtype adult MF1 male mice treated with vehicle or COUMATE (10mg/kg, p.o.).** Of the six candidate genes identified as being differentially expressed in the microarray analysis, none showed differential expression between the drug and vehicle-treated groups.
